# Bacterial infections among patients with psychiatric disorders: Relation with hospital stay, age, and psychiatric diagnoses

**DOI:** 10.1371/journal.pone.0208458

**Published:** 2018-12-04

**Authors:** Michael Belz, Nico Rehling, Ulrike Schmidt, Jens Wiltfang, Bernhard Kis, Claus Wolff-Menzler

**Affiliations:** 1 Department of Psychiatry and Psychotherapy, University Medical Center Göttingen (UMG), Göttingen, Lower-Saxony, Germany; 2 German Center for Neurodegenerative Diseases (DZNE), Goettingen, Lower-Saxony, Germany; 3 Institute for Biomedicine (iBiMED), Medical Sciences Department, University of Aveiro, Aveiro, Portugal; University of Toronto, CANADA

## Abstract

The prevalence of infections is supposed to be higher in older patients and to extend the length of hospital stays. This study aimed, first, to test this supposition within a large psychiatric population which we divided into four clusters of psychiatric ICD-10 diagnoses: F00-F03 (dementias), F10 (substance disorders), F20-29 (schizophrenia, schizophreniform and other non-mood psychotic disorders), F32-F33 (major depressive disorders). Second, despite the increasing evidence for the role of infections in psychiatric disorders, it is, to the best of our knowledge, largely unknown whether the rates of infections with pathogens of the four most frequent germ families differ between psychiatric diseases. Thus, in a retrospective study, the results of clinical routine examinations (pap smear, analysis of midstream urine, stool) dependent on symptoms in 8545 patients of a German psychiatric clinic were analyzed in a 12-year dataset. Results show that a longer hospital stay was associated with an increased number of microbiological tests, but led to no significant difference between positive vs. negative findings. Consistent with previous studies, patients with infections were older than patients without infections. For the F10 diagnosis cluster we found a significantly reduced (F10: *Staphylococcaceae*) and for the F20-29 cluster a heightened risk of infections (*Staphylococcaceae*, *Corynebacteriaceae*). Furthermore, patients belonging to the F00-F03 cluster exhibited elevated rates of infections with all four germ families. The latter can be ascribed to patients’ age as we found higher age to be associated with these infections, independently of the presence of dementia. Our results suggest that different psychiatric diagnoses are associated with a heightened or lowered risk of bacterial infections and, furthermore, that clinical routine infection-screenings for elderly psychiatric patients seems to be reasonable.

## Introduction

Patients’ infections during hospital stay go along with multiple negative consequences such as a heightened psychological strain, (due to) additional diagnostic examination and medical treatment [1,2]. A connection of bacterial infections and a prolonged and hence more costly [3] duration of the hospital stay was reported repeatedly, both for semi-residential and residential treatment [4–6]. Generally, the risk of infections rises with higher age, most likely due to a less efficient immune system [7]. There is increasing evidence for a role of specific bacterial taxa in psychiatric disorders [8].

Here, to enable a systematic comparison of bacterial infections between patients with different F-diagnoses according to ICD-10, patients were clustered into three groups of F-diagnoses which comprise the top three psychiatric diagnoses [9], on which we aimed to focus in this study: (1) Unipolar depression (F32-F33), (2) mental and behavioral disorders due to use of alcohol (F10), and (3) schizophrenia, schizotypal and delusional disorders (F20-F29, including diagnoses which are clinically/etiologically related to schizophrenia). Due to the increasing risk of bacterial infections with rising age and due to the prognosis that the prevalence of neurodegenerative diseases will drastically increase within the next 30 years due to the aging population [10], we also included dementia in our analyses (F00-F03).

In literature, an increasing prevalence of both bacterial and viral infections, have been described for patients with psychiatric disorders. Studies reporting on the four clusters of diagnoses we study here so far resulted in heterogeneous results: (1) In their meta-analysis of 6362 studies, Wang et al. showed significant associations between the presence of major depression and heightened infection rates with multiple viral, (e.g., *Borna*, *Herpes-simplex*, *Varicella-Zoster*, *Epstein-Barr*), as well as a bacterial pathogen (e.g., *Chlamydophila trachomatis*) [11]. (2) Alcohol abuse generally impairs the immune response [12] thereby heightening the risk for severe infectious diseases such as tuberculosis [13] and community-acquired pneumonia [14]. (3) For schizophrenia and related disorders, viral infections were reported (e.g., *Herpes simplex*, *Epstein-Barr*, *Arboviruses*, *Polio*) [15], as well as infections with *Toxoplasma gondii* and *Borrelia* [16]. For patients with Alzheimer’s disease, studies showed a heightened risk for bacterial infections with spirochetes and *Chlamydophilia pneumoniae* [17,18].

Against the background of these heterogeneous results, this study focuses on bacterial infections and their association with hospital stay, age, and selected clusters of psychiatric diagnoses. To our knowledge, we are the first to perform a systematic analysis of these variables in a psychiatric population (*N* = 8545). Specific associations between bacterial infections and psychiatric diagnoses can help to improve the clinical risk assessment, e.g., to define the probability for patients with neurodegenerative disease to suffer from (formerly undetected) infections–thereby stressing the necessity to intensify screening procedures. Using a 12-year-routine-dataset of a psychiatric hospital, we focused on the following questions:

- (1) Are patients with bacterial infections comparably older and do they show a prolonged hospital stay as described in the literature?
- (2) Does this pattern also apply to the four clusters of diagnoses studied here?
- (3) Can relative over- or under-representations of the four most frequent germ families be identified within the clusters of diagnoses?
- (4) In patients suffering from dementia: In case the dataset at hand also reveals a relatively heightened prevalence of bacterial infections in patients with neurodegenerative disorders: Does the risk of infection rise due to pathological neurodegeneration or due to physiological aging?

## Materials and methods

### Data acquisition

In a retrospective study, a 12-year-routine-dataset in pseudonymous form was created in cooperation with the Data-Resource-Center of the University Medical Center Göttingen (UMG) and the Institute for Medical Microbiology. The dataset contains data of all patients admitted to the Department of Psychiatry and Psychotherapy between 2004-01-01 and 2015-12-31. The dataset was analyzed as part of an ongoing thesis project at the University Medical Center Göttingen. Diagnoses were determined in clinical expert interviews during clinical routine examinations. We excluded relatives, outpatients, and patients without an ICD-10 psychiatric diagnosis, or treatment beyond 2015-12-31. To reduce the systematic bias due to repeated hospital stays, each patient was only included once, namely during his/her first treatment. The study was approved by the Local Ethics Committee of the University Medical Center Göttingen (application number DOK_45_2015). Due to anonymized and retrospective data analysis, no form of consent was obtained.

We included patients who were treated in the Department of Psychiatry and Psychotherapy between 2004-01-01 and 2015-12-31. In accordance with the treatment guidelines of the University Psychiatric Clinic Göttingen, patients were tested for infections upon occurrence of symptoms pointing to an infection. When infections were suspected, bacteriological analyses were performed in midstream urine (52.4%), pap smear (25.3%), stool (6.6%), and urine samples from permanent catheter (5.9%). Other types of samples comprised < 5%. If multiple bacteriological tests were conducted during the first hospital stay, only the first test was included to avoid a possible over-representation and addition of a confounding factor (heightened infection risk due to length of hospital stay). If multiple tests were conducted, results were included as multiple positive.

In this study, the following patient-related data was included in the final dataset: (1) demographic variables (age, gender), (2) duration of hospital stay, (3) results of the microbiological testing, (4) main diagnosis (see S1 Dataset).

### Statistical analyses

Data was analyzed using IBM SPSS Statistics 25. For presentation, sum scores, means (*M*) and standard deviations (±) were created. We chose different analytic approaches, to answer each of the four research questions (see introduction). First, to analyze general differences between patients, two UNIANOVAs with a three-stage between-individual factor (no test vs. negative test result vs. positive test result) were created for the dependent variables (1) hospital stay in days and (2) age in years, thus focusing on the question if patients with bacterial infections were comparably older and if they showed a prolonged hospital stay. Second, the four clusters of diagnoses (F32-F33, F10, F20-29, F00-F03) were included as additional between-individual factor in both models. Therefore we searched for a possible connection between bacterial infections and higher age/prolonged hospital stay in each cluster of diagnoses. Third, to analyze if the clusters of diagnoses were associated with the four most frequent germ families in terms of relative over- or under-representations of the latter, four Chi-Square-Tests, each containing a 5×2-matrix (four clusters of diagnoses plus the cluster “other diagnose” × two states of testing: positively vs. negatively/not tested) and corresponding odds ratios, were calculated. Fourth, to eliminate age as confounder for a possible heightened prevalence of bacterial infections in patients with neurodegenerative disorders, an age-matched subsample was created to correct all corresponding odds ratios. All levels of significance are reported two-tailed. All *p*-values were corrected for the number of statistical tests according to the Bonferroni-method, to prevent α-error inflation.

## Results

### Subjects, diagnoses, and germ families

A total of *N* = 8545 patients, 50.3% female (*n* = 4296) and 49.7% male (*n* = 4249), were included in the study. Of the total sample, 11.4% (*n* = 978) were treated semi-residentially. The mean age was *M* = 45.905 ± 17.519 years, the mean hospital stay lasted *M* = 28.228 ± 32.498 days (age and hospital stay correlated by *r* = 0.03, Pearson correlation coefficient < 0.10: minimal to none effect [19]). In sum, 314 different main diagnoses were given. The five most frequent diagnoses were (1) F10.2 (10.9%, *n* = 929), (2) F33.2 (9.2%, *n* = 788), (3) F32.2 (8.7%, *n* = 744), (4) F20.0 (6.5%, *n* = 556), and (5) F43.2 (4.6%, *n* = 396). Overall, *n* = 7818 (91.5%) patients were not tested for bacterial infections, *n* = 351 (4.1%) had a negative result, and *n* = 376 (4.4%) were positively tested.

Due to the high number of different main psychiatric diagnoses, four diagnosis clusters were built based on the ICD-10-GM-2017 catalogue (see introduction). The following clusters, ordered by numbers, were included: (1) Unipolar depressions (F32-F33, *n* = 2553, 29.9%); (2) mental and behavioral disorders due to use of alcohol (F10, *n* = 1200, 14.0%); (3) schizophrenia, schizotypal and delusional disorders (F20-F29, *n* = 1016, 11.9%); (4) dementia (F00-F03, *n* = 342, 4.0%, also including G30.0, G30.1, G30.8, G30.9, and G31.82). Additionally, the cluster (5) “other” (*n* = 3434, 40.2%) was defined.

During microbiological testing, a total of 56 different types of bacterial infections in *n* = 376, partially multiple-positive patients, were found. To render statistical analysis possible, infections were grouped according to the four most frequent germ families according to their species and taxonomic classification, as defined by the “list of prokaryotic names with standing in nomenclature” (LPSN, [20]): (1) *Enterobacteriaceae* (*n* = 169, 44.9%); (2) *Staphylococcaceae* (*n* = 149, 39.6%); (3) *Enterococcaceae* (*n* = 91, 24.2%); (4) *Corynebacteriaceae* (*n* = 46, 12.2%).

### Bacterial infection and hospital stay

**Fig 1:**
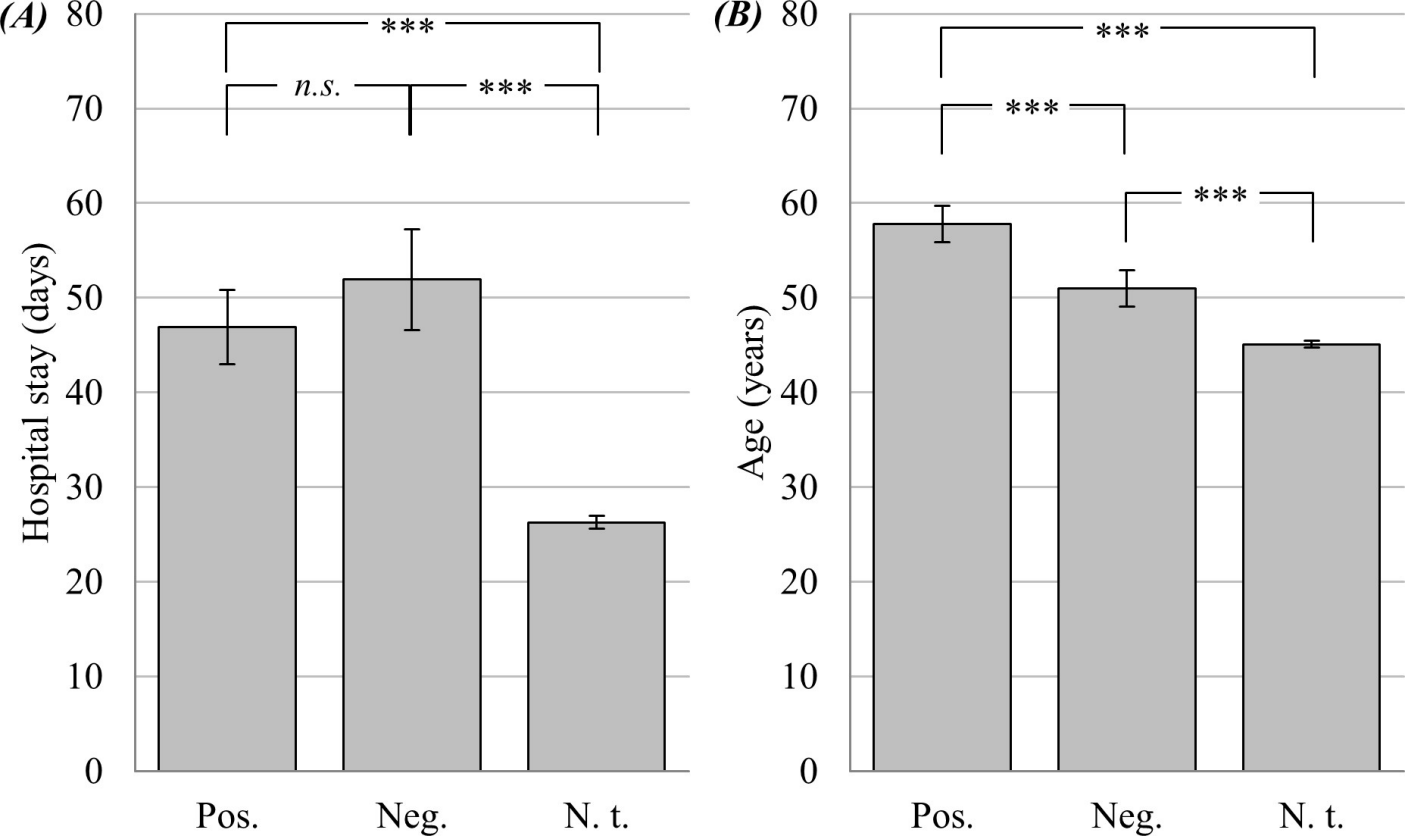
Hospital stay and age. * *p* < .05; ** *p* < .01; *** *p* < .001. Means (*A* = hospital stay in days; *B* = age in years) with 95%-CI and Bonferroni-corrected pairwise comparisons, separated by microbiological testing for bacterial infections: Pos. (positive result, *n* = 376), Neg. (negative result, *n* = 351), N. t. (no test, *n* = 7818), *N* = 8545.

The UNIANOVA revealed significant variation of hospital stay (UNIANOVA: *F*(2, 8530) = 106.437, *p* < .001, Fig 1A), which was then further analyzed by post hoc tests: While the difference between positively- (*M* = 46.923 ± 38.753) and negatively-tested patients (*M* = 51.920 ± 50.602) missed significance (*p* = 0.086), patients who were not tested had the significantly shortest hospital stay (*M* = 26.266 ± 30.361, *p* < .001 for both pairwise comparisons).

**Fig 2:**
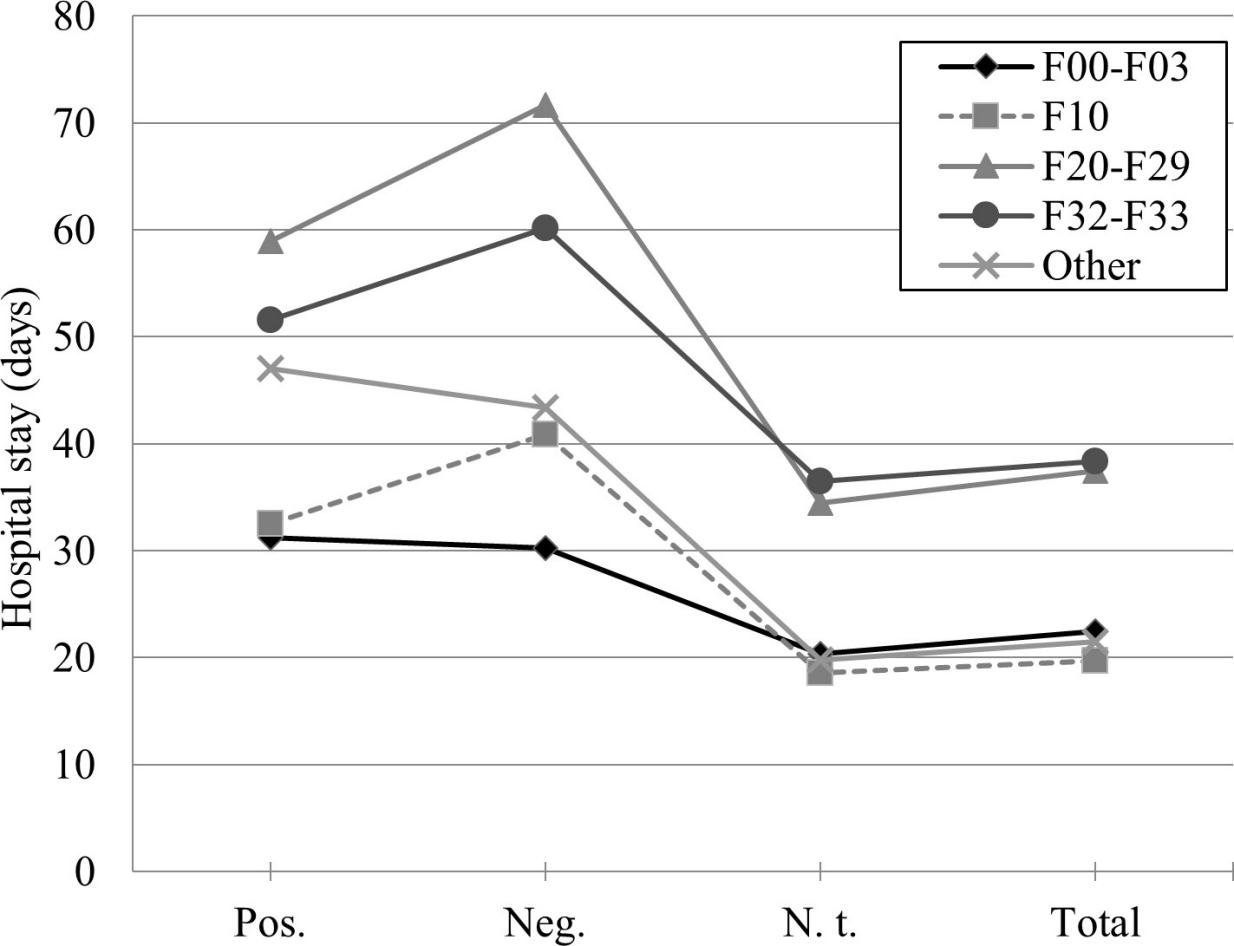
Hospital stay and clusters of diagnoses. Mean hospital stay of patients in days for each cluster of diagnoses, separated by microbiological testing for bacterial infections: Pos. (positive result, *n* = 376), Neg. (negative result, *n* = 351), N. t. (no test, *n* = 7818), Total (total sample, *N* = 8545).

Also, the UNINANOVA showed differences between the clusters of diagnoses concerning the length of the hospital stay (*F*(4, 8530) = 34.750, *p* < .001, Fig 2). For every cluster, the shortest hospital stay was found in patients who were not tested. For the total sample, we found the longest hospital stay for the cluster (1) F32-F33 (*M* = 38.349 ± 36.670), followed by (2) F20-F29 (*M* = 37.459 ± 37.333), (3) F00-F03 (*M* = 22.500 ± 19.234), and (4) F10 (*M* = 19.704 ± 22.043). Concerning the cluster “other diagnoses”, the hospital stay was *M* = 21.522 ± 28.902 days. In four out of 10 corrected pairwise comparisons, significance was missed (*p* between .78 and 1.00): (1) “other diagnoses” vs. F00-F03, (2) “other diagnoses” vs. F10, (3) F00-F03 vs. F10, and (4) F20-F29 vs. F32-F33. The remaining six pairwise comparisons revealed significant differences between the clusters of diagnoses (all *p*-values < .001).

### Bacterial infection and age

Analyses showed that age varied significantly between positively-, negatively-, and not-tested patients (UNIANOVA: *F*(2, 8530) = 42.812, *p* < .001, Fig 1B). Positively-tested patients (*M* = 57.801 ± 19.165) were significantly older than negatively-tested (*M* = 51.022 ± 18.328, *p* < .001), as well as not-tested patients (*M* = 45.103 ± 17.151, *p* < .001). Additionally, the difference between negatively- and not-tested patients was significant (*p* < .001).

**Fig 3:**
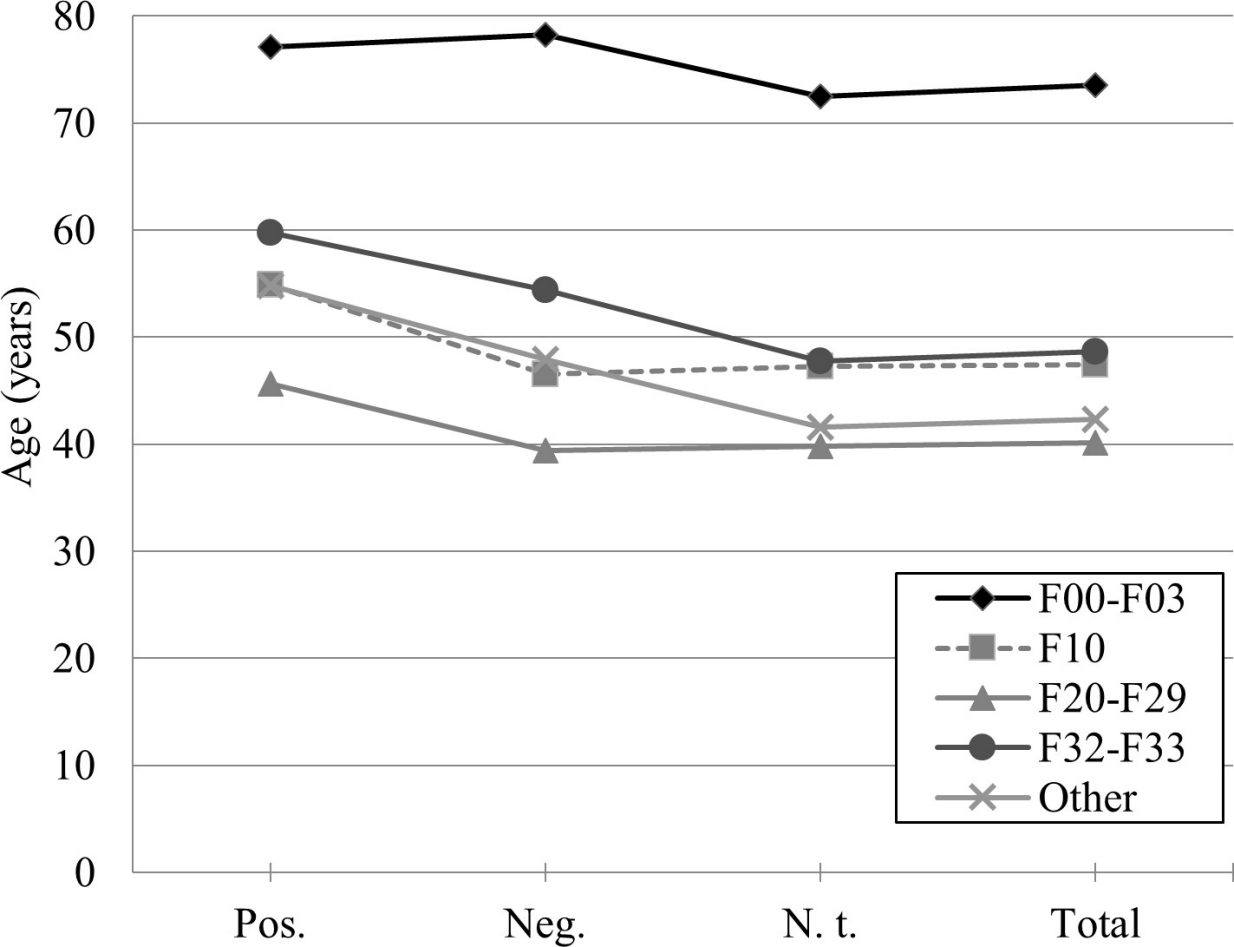
Age and clusters of diagnoses. Mean age of patients in years for each cluster of diagnoses, separated by microbiological testing for bacterial infections: Pos. (positive result, *n* = 376), Neg. (negative result, *n* = 351), N. t. (no test, *n* = 7818), Total (total sample, *N* = 8545).

Age varied significantly between the clusters of diagnoses (UNIANOVA: *F*(4, 8530) = 108.223, *p* < .001, Fig 3). For the total sample, we found that patients belonging to the cluster (1) F00-F03 showed the highest age (*M* = 73.512 ± 10.196), followed by (2) F32-F33 (*M* = 48.641 ± 16.909), (3) F10 (*M* = 47.468 ± 12.367), and (4) F20-F29 (*M* = 40.120 ± 15.027). The mean age within the cluster “other diagnoses” was *M* = 42.288 ± 17.761 years. Only the difference between the clusters F10 and F32-F33 missed significance (*p* = .373). All remaining pairwise comparisons between the clusters of diagnoses revealed significant results for age (*p* between .002 and < .001 in nine out of 10 corrected pairwise comparisons).

### Clusters of diagnoses and germ families

The results of the analyses are summarized in Table 1. In each 5×2 matrix, significant deviations between expected and observed frequencies for the four germ families were present in the clusters of diagnoses (*p* between .027 and < .001).

**Table 1:** Bacterial infections, arranged by germ families and clusters of diagnoses.

Diagnoses	∑	∑_pos._	∑_≠pos._	Odds ratio	χ^2^_(df = 4)_	*p*(χ^2^)^a^
**(A)** *Enterobacteriaceae*						
1. All pat.	8545	169	8376			
2. F00-F03	342	21 (12.4%)	321 (3.8%)	3.561*	35.842***	< .001
3. F10	1200	17 (10.1%)	1183 (14.1%)	0.680		
4. F20-F29	1016	24 (14.2%)	992 (11.8%)	1.232		
5. F32-F33	2553	52 (30.8%)	2501 (29.9%)	1.044		
6. Other diagnose	3434	55 (32.5%)	3379 (40.3%)	0.713*		
**(B)** *Enterococcaceae*						
1. All pat.	8545	91	8454			
2. F00-F03	342	11 (12.1%)	331 (3.9%)	3.374*	16.916*	= .016
3. F10	1200	9 (9.9%)	1191 (14.1%)	0.669		
4. F20-F29	1016	10 (11.0%)	1006 (11.9%)	0.914		
5. F32-F33	2553	29 (31.9%)	2524 (29.9%)	1.099		
6. Other diagnose	3434	32 (35.2%)	3402 (40.2%)	0.805		
**(C)** *Corynebacteriaceae*						
1. All pat.	8545	46	8499			
2. F00-F03	342	6 (13.0%)	336 (4.0%)	3.644*	15.762*	= .027
3. F10	1200	6 (13.0%)	1194 (14.0%)	0.918		
4. F20-F29	1016	10 (21.7%)	1006 (11.8%)	2.069*		
5. F32-F33	2553	12 (26.1%)	2541 (29.9%)	0.828		
6. Other diagnose	3434	12 (26.1%)	3422 (40.3%)	0.524		
**(D)** *Staphylococcaceae*						
1. All pat.	8545	149	8396			
2. F00-F03	342	16 (10.7%)	326 (3.9%)	2.978*	31.226***	< .001
3. F10	1200	10 (6.7%)	1190 (14.2%)	0.436*		
4. F20-F29	1016	27 (18.1%)	989 (11.8%)	1.657*		
5. F32-F33	2553	49 (32.9%)	2504 (29.8%)	1.153		
6. Other diagnose	3434	47 (31.5%)	3387 (40.3%)	0.681*		

Diagnoses = clusters of diagnoses; ∑ = number of patients; ∑_pos._ = number of positively tested patients (germ families A, B, C, D); ∑_≠pos._ = number of negatively/not tested patients; Odds ratios (each corresponding cluster of diagnoses was tested within the germ families A, B, C, D vs. the remaining sample; * 95%-CI ≠ 1); χ^2^: empirical Chi-Square for each 5x2 matrix (*df* = 4, *N* = 8545); *p*(χ^2^)*: p-value for each Chi-Square-Test.

^a^ * *p* < .05

** *p* < .01

*** *p* < .001.

Regarding the odds ratios, specific higher/lower chances between clusters of diagnoses and germ families were identified (see Table 1): While the cluster F00-F03 showed a significantly heightened relative prevalence of all four germ families, a reduced probability of *Staphylococcaceae* was found in cluster F10. In cluster F20-F29, there was a significantly heightened relative prevalence of *Corynebacteriaceae* and *Staphylococcaceae*. Analyses of cluster F32-F33 showed no alteration in the relative prevalence for any of the four germ families.

### Influence of age on patients’ vulnerability towards bacterial infections

As expected, patients with dementia showed a higher age than other subgroups (*n* = 342, *M* = 73.512 ± 10.196, *r* = 0.32, Fig 3). Moreover, older patients had a higher relative prevalence of bacterial infections (see Fig 1B, Table 1). We controlled for a possible effect of age by comparing two age-matched samples of which one included and the other excluded patients suffering from the diagnoses F00-F03 (age ≥ 66 years; *n* = 1037, *M* = 73.397 ± 6.132). Results show, that patients with F00-F03 and patients without did not differ significantly in their odds ratios (odds ratios: 0.898 to 1.834, *n*.*s*., see Table 2 for an overview). In summary, high age but not dementia diagnoses was identified as possible confounder for heightened infection rates in patients with dementia.

Analogous to the cluster F00-F03, we calculated correlations between each cluster of diagnoses and age. Results show only minimal to none correlation [19] between age and F10 (*r* = 0.04), F20-F29 (*r* = -0.12), as well as F32-F33 (*r* = 0.10). We thus did not generate age-matched samples for the remaining clusters, as we found age to be a relevant confounder for the cluster F00-F03 only (Table 2).

**Table 2:** Comparison between F00-F03 and an age-matched sample.

Diagnoses	∑	∑_pos._	∑_≠pos._	Odds ratio
**(A)** *Enterobacteriaceae*				
1. All pat.	1379	79	1300	
2. F00-F03	342	21 (26.6%)	321 (24.7%)	1.104
3. ≠ F00-F03	1037	58 (73.4%)	979 (75.3%)	
**(B)** *Enterococcaceae*				
1. All pat.	1379	48	1331	
2. F00-F03	342	11 (22.9%)	331 (24.9%)	0.898
3. ≠ F00-F03	1037	37 (77.1%)	1000 (75.1%)	
**(C)** *Corynebacteriaceae*				
1. All pat.	1379	16	1363	
2. F00-F03	342	6 (37.5%)	336 (24.7%)	1.834
3. ≠ F00-F03	1037	10 (62.5%)	1027 (75.3%)	
**(D)** *Staphylococcaceae*				
1. All pat.	1379	62	1317	
2. F00-F03	342	16 (25.8%)	326 (24.8%)	1.057
3. ≠ F00-F03	1037	46 (74.2%)	991 (75.2%)	

Diagnoses = clusters of diagnoses; ∑ = number of patients; ∑_pos._ = number of positively tested patients (germ families A, B, C, D); ∑_≠pos._ = number of patients with negative test results/not tested patients; Odds ratios (F00-F03 was tested for the germ families A, B, C, D vs. an age-matched sample without F00-F03 (≠ F00-F03); * 95%-CI ≠ 1).

## Discussion

In our study, we investigated bacterial infections within a psychiatric hospital, and their relation with hospital stay, age, and F-diagnoses.

In contrast to the findings of multiple other studies [4–6], in our psychiatric population a longer hospital stay was not associated with positive microbiological findings concerning bacterial infections. Instead, a longer hospital stay was associated with a higher probability to be *generally tested* for bacterial infections. This elevated number of tests in patients who stayed longer in hospital did not produce a bias for positive microbiological testing results, but instead resulted in almost equally distributed positive *and* negative findings, as shown in Fig 1A (pos. vs. neg., *n*.*s*.). In sum, patients whose microbiological tests indicated an acute bacterial infection did not stay longer in hospital compared to patients who were tested negatively. Furthermore, patients who were not tested at all showed the shortest hospital stay. A possible explanation would be that along with an enduring hospital stay, the probability of being tested for a bacterial infection rises. However, as our results clearly show, a comparably higher testing frequency did not produce a bias for positive testing results.

In accordance with literature [7], we detected a heightened risk for bacterial infections with increasing age in this study: Patients with positive test results were significantly older than patients with negative results and patients who were not tested at all. Except for minimal deviations, this pattern was consistent across all clusters of diagnoses. Interestingly, we also found negatively tested patients to be significantly older than patients who were not tested. One possible explanation is that physicians tend to test older patients more often for infections due to expected vulnerability (see above). As we found, this may lead to an increase in age for positively tested, but coevally for negatively tested patients, as shown in Fig 1B.

Relative over- and under-representations of the four most frequent germ families could be identified within multiple clusters of diagnoses. Patients who were diagnosed with F10 had a significantly reduced chance to get infected with *Staphylococcaceae* during hospital stay. So far, this has been reported only for *Bacteroidaceae* [21] and has been explained by a reduced connectivity of the mucosa by the authors. Furthermore, patients who were diagnosed with F20-F29 were infected more often with *Corynebacteriaceae* and *Staphylococcaceae*. This has not been described in the literature so far–instead, heightened infection rates with *Chlamydiaceae* (*Chlamydophilia pneumoniae*, *Chlamydia trachomatis*) have been described [22,23]. Despite an extensive literature research, we only found few explanatory models to describe connections between bacterial infection and psychiatric disorders. Thus, the partial relative over- and under-representations of bacterial infections we found in this study for patients diagnosed with F10 and F20-29 cannot be interpreted, but–to this point–only be described.

Patients who were diagnosed with dementia (cluster F00-F03) were, as expected, older than the other subgroups. Overall, these patients showed the highest risk for bacterial infections which was significantly elevated for all four germ families. This relatively heightened prevalence could be controlled by comparing the cluster F00-F03 with an age-matched sample, without a diagnosis of dementia: Compared to a subgroup of patients at the same age, but without the diagnoses F00-F03, we did not find any significant differences in terms of bacterial infections. We conclude that age seems to be the main reason for the high infection risk for the subgroup F00-F03, not the diagnosis of dementia.

### Limitations

(1) The generalizability of results which base on a 12-year-routine-dataset in pseudonymous form is limited *per se*, due to the absence of experimental manipulation and due to time as confounding factor (repeated hospital stays, mutual infections). Thus, the analyses of this cross-sectional study focus on the description of the current state (e.g., positive tests for infection rates were related to higher age). Clearly, causal inferences cannot be drawn from this routine dataset: we found that single clusters of diagnoses show relative over- or under-representations of germ families whereas specific explanations for these deviations have to be identified in future studies. A longitudinal approach, which was impossible to perform in the routine dataset analyzed in the study presented here, would enable e.g., the analysis of chronological sequences of infections. (2) The initial sample size can be described as huge (*N* = 8545), but the number of *n* = 376 patients with infections over a time span of 12 years is relatively small. Inclusion of further routine-datasets from other hospitals would enable to analyze additional subgroups (e.g., semi-residential vs. residential, more clusters of diagnoses, differentiation within F00-F03, etc.). (3) Due to the usage of routine data in this study, a number of un- or misclassified infections can be anticipated. This limits the interpretability of our results by reason of possible biases: e.g., for the subsample of not-tested patients, infections may have been overlooked due to low symptom expression. One possibility to reduce error rates would be the future optimization of the screening procedure including (more) supervision by experienced peers.

## Conclusions

We found only a few studies focusing on bacterial infections within populations suffering from psychiatric disorders. This study provides first results which contribute to elucidation of a (so far) not sufficiently explored and understood, but visible relationship between age, infections and psychiatric disorders: Here, we identified a relative underrepresentation of *Staphylococcacaeae* for the diagnosis cluster F10 and relative overrepresentations of *Corynebacteriaceae* and *Staphylococcaceae* for F20-F29. In the future, the analysis of multicenter-datasets will increase the sample size thereby possibly allowing the analysis of smaller diagnostic entities, ideally, of single diagnostic entities, instead of clusters. And, furthermore, this might allow for further taxonomic differentiation of germ families. In the long term, this research might help to identify bacterial infections as possible etiopathogenetic factor or, at least, marker of psychiatric disorders or distinct psychopathological conditions.

The high vulnerability of older patients for bacterial infections leads to the conclusion that–independently of a diagnosed dementia–intensifying screening procedures for geriatric patients could reduce the health risk for themselves and others.

## Supporting information

S1 Dataset Dataset of the study. The minimal dataset of the study is provided as SAV-file (SPSS).
(SAV)
